# Steady flow, not steady state – a plea for physiological thinking

**DOI:** 10.1007/s00709-021-01673-7

**Published:** 2021-06-12

**Authors:** Peter Nick

**Affiliations:** Botanical Institute, Karlsruhe Institute of Technology, Karlsruhe, Germany

When Boris Belousov, in the attempt to mimick the Krebs Cycle in a very simplified test-tube system based on citric acid and cerium, discovered that this system started to oscillate, he had a hard time of fighting over an entire decade with rejective journals (for review see Winfree 1984). In the meantime, the Belousov-Zhabotinsky Oscillator is often used as model for the fact that life acts far from thermodynamic equilibrium. Depending on the details of the set-up, these oscillations can become manifest either in time (as rhythmical changes of colour), or in space (as patterns). Despite its fascination and beauty, this chemical system represents probably more a metaphor rather than a true model for life. Nevertheless, there are fascinating facets that can be extracted to understand biological phenomena. Oscillations differ from the steady state prevailing in chemistry because they harbour an element of inertia. There is noticeable *delay*, after a state dissipates, before the next state is established. In other words: As it is the case for living systems, also the Belousov-Zhabotinsky Oscillator is subject to *time*, it has a history of becoming. If we really want to understand living systems, it is not sufficient to consider the states, but also the transitions between them.

Three contributions to the current issue are doing exactly this – they scrutinise the transitions and arrive at surprising insights into the way, how cells and how organisms work. Ironically, all three examples are dealing with a life form that is usually mis-understood as rather static: plants.

The first contribution to the current issue, by Sommer et al. (2021), addresses membrane flow in *Chara*, a green alga with giant internodal cells that has been a classical system of plant cell biology, because due to their size, these cells are amenable to experimental manipulation. Since *Chara* still has remained recalcitrant to genetic manipulation, it has at the turn of the century, become out of fashion as a model. However, during recent years, these organisms have attracted considerable attention as sister lineage to the early land plants (Becker 2013). And as the authors demonstrate, its suitability for cell biology can compensate some of the genetic drawbacks. They use a pulse-chase experiments with two classes of fluorescent membrane dyes to investigate the details of endocytosis. Here, the fluorescent hydrazides behaved differently from the widely used styryl dyes. In particular, the uptake of the hydrazides was not inhibited by cytochalasin D, a drug efficiently eliminating the fine actin meshwork subtending the membrane, while short thick actin rods develop in Chara (Foissner and Wasteneys 2000). Moreover, these dyes accumulated in so called charosomes, specific convolutions of the plasma membrane that might be relevant for repair under conditions of perturbed membrane integrity (Franceschi and Lucas 1980). This was then experimentally addressed and confirmed inflicting local wounds by a strong laser. Thus, using different fluorescent markers, authors succeeded to detect two differential routes of endocytotic uptake, one which was part of steady-state membrane dynamics, and a second one, which was not part of this steady state, but accumulated a reservoir to be able to respond to situations, when the steady state is challenged by perturbations.

The contribution by Tazawa et al. (2021) to the current issue is addressing the problem of flow on a biophysical level, by the way in the same experimental system, the internodal cells of *Chara*. While biological membranes are said to be semipermeable (which is essentially correct), the ability to allow water permeation does not mean that it is sufficient to describe cellular behaviour merely in terms of water potential gradients. The hydraulic resistance of membranes is often limiting, and can cause significant problems, when cells face sharp differences in water potential. The authors have been working on membrane behaviour in the context of growth physiology for many decades – the primordial work, by the way also published in this journal, dates by to 1956 (Kamiya and Tazawa 1956). In the current work, they measure hydraulic resistance of the cell as entity, but as well as that of plasma membrane and tonoplast. As variable they use cell age because all membranes become less water permeable with age. They find that the hydraulic resistance of the tonoplast is much lower than that of the plasma membrane, which means that vacuole and cytoplasm act as one osmotic unit. This allows to buffer volume changes of the cytoplasm under osmotic challenge, such that cytoplasmic architecture and homeostasis are preserved. The difference in the hydraulic resistances decreases with cell age, which is probably due to a decreasing abundance of aquaporins. The physiological consequences are probably compensated by the concomitantly thickening cell wall. The beauty of this work is in the integration of a biophysical parameter (hydraulic conductivity of membranes) into growth and development of an organism. During the growth phase of the cell, the ability for dynamic flow is carefully regulated – at a later phase, when growth has been completed, the regulation of dynamic flows is less crucial, here the control of the steady state is sufficient.

The third contribution, by Duarte et al. (2021) extends analysis of flows to the organismic level, addressing oxygen flow in amphibic plants. These plants are often endowed with air-filled conductive tissues, so called aerenchyma to provide gas exchange to the submersed organs. The Reedmace or Bulrush (*Typha*) is a grass, recapitulating the amphibian lifestyle of the earliest vascular landplants with a sturdy rhizome that can thrive (and breathe) in the mud, i.e., in a mostly anoxic environment. Since the partial oxygen pressure in the surrounding medium is low, the oxygen that is entering the rhizome through the aerenchyma is rapidly lost through radial diffusion. By comparing intact plants with plants, where the leaves had been partially or entirely removed, to modulate the oxygen import into the rhizome, the authors analysed the adaptive responses in the rhizome. They found that the differentiation into aerenchyma was stimulated by oxygen deprivation including tissue differentiations such as the formation of diffusion barreers, including suberinised interfaces. Motivated by the idea that a part of the oxygen is not imported from the aerial parts, but generated in site by dissipation of hydrogen peroxide, the authors conducted experiments, where catalase in the rhizome was either stimulated or inhibited. In fact, they could demonstrate that catalase accounts for a major part of the oxygen steady-state levels and, thus, complements oxygen import from the aerial parts.

All three contributions address processes rather than states. This is, in fact, the approach of physiology. Compared to the genetic and -omics strategies often prevailing in biology, it may seem outdated to do physiology. However, we should keep in mind that collecting genes and molecules, as important it is to attain a molecular understanding of a phenomenon, need to be complemented by process based, physiological, analysis. To look at the steady state, will give us a snapshot. If we want to see the entire movie, we need to look at steady flow between these steady states. To *become* is as important in biology as to *be*.
